# Assessment of implementation of antibiotic stewardship program in surgical prophylaxis at a secondary care hospital in Ras Al Khaimah, United Arab Emirates

**DOI:** 10.1038/s41598-020-80219-y

**Published:** 2021-01-13

**Authors:** Hessa Saleh Alshehhi, Areeg Anwer Ali, Duaa Salem Jawhar, Essam Mahran Aly, Srinivas Swamy, Manal Abdel Fattah, Khawla Abdullah Drweesh, Azzan Alsaadi

**Affiliations:** 1 Department of Clinical Pharmacy and Pharmacology, Rak College of Pharmaceutical Sciences, RAK Medical and Health Sciences University, Ras Al Khaimah, United Arab Emirates; 2 Pharmacy Department, Saqr Hospital, Ministry of Health and Prevention, Ras Al Khaimah, United Arab Emirates; 3 Anesthesia Department, Saqr Hospital, Ministry of Health and Prevention, Ras Al Khaimah, United Arab Emirates; 4 Surgical Department, Saqr Hospital, Ministry of Health and Prevention, Ras Al Khaimah, United Arab Emirates; 5 Pure Health Company, Dubai, United Arab Emirates; 6 Pediatric Department, Saqr Hospital, Ministry of Health and Prevention, Ras Al Khaimah, United Arab Emirates; 7 Present Address: Pharmacy Department, Abdullah Bin Omran Hospital for Obstetrics & Gynecology, Ministry of Health and Prevention, Ras Al Khaimah, United Arab Emirates

**Keywords:** Diseases, Health care, Medical research

## Abstract

Antibiotic overuse is a major factor for causing antibiotic resistance globally. However, only few studies reported the implementation and evaluation of antimicrobial stewardship programs in Gulf Cooperation Council. This study was conducted within 8-months periods to evaluate the effect of the newly implemented antibiotic stewardship program on improving the prescribing practice of surgical antibiotic prophylaxis in a secondary care hospital in the United Arab Emirates by releasing local hospital guidelines. The data of 493 in patients were documented in the predesigned patient profile form and the prescribing practice of surgical antibiotic prophylaxis for clean and clean-contaminant surgical procedures was compared and analyzed two months’ prior (period A) and post (period B) the implementation of antibiotic stewardship program. The 347 patient’s data (PD) were analyzed during period A and 146 PD during period B. The prescription of piperacillin/tazobactam was decreased from 2.4% from all surgical prophylaxis antibiotic orders in period A to 0% in period B. The appropriateness of the antibiotic therapy was found to differ non significantly for the selection of prophylactic antibiotic (*p* = 0.552) and for the timing of first dose administration (*p* = 0.061) between A and B periods. The total compliance was decreased non significantly (*P* = 0.08) from 45.3 to 40.2%. Overall, the guidelines have improved the prescribing practice of antibiotics prior to surgery. However, further improvement can be achieved by initiating educational intervention via cyclic auditing strategy.

## Introduction

Over usage of antibiotics especially the broad spectrum ones, and non-compliance with infection prevention and control measures in hospital setting results in developing nosocomial infections involving antimicrobial resistant strains. This might contribute further to the spread of multidrug resistant organisms worldwide^1,2^. Antimicrobial resistance (AMR) poses a constant challenge to the use of antibiotics in the Middle East and Gulf regions due to the limited availability of reliable data regarding the resistant organisms and antibiotics utilization pattern^1,3^. In the Gulf region, the Gulf Cooperation Council Center for Infection Control (GCC-IC) has developed a strategic plan in 2014 to be adopted by GCC countries in order to implement country-based plans for combating AMR^4^. Since its establishment in 2010, the UAE’s Antimicrobial Resistance Surveillance System (AMRSS) has played a significant role in collection and reporting of data from the different healthcare facilities across the seven emirates of the country. According to antimicrobial resistance surveillance in Abu Dhabi, United Arab Emirates (UAE), there is a high prevalence of multidrug-resistance pathogens, associated with increasing trends of resistance^1,5–7^. In addition, the antimicrobial stewardship programs (ASPs) were recently introduced in the UAE hospitals, with the aim of improving the quality of usage of antimicrobial medications, thereby, enhancing patient health outcomes and reducing the emergence of resistance^8–11^.

One of the major aspects of ASPs is the effective and rational use of surgical antimicrobial prophylaxis (SAP) prior to surgical procedures^5^. Literature data on utilization of SAP in UAE hospitals are limited^9–13^. Hence, the current study was carried out in a secondary care hospital in Ras Al Khaimah to assess the implementation of newly designed and introduced local hospital surgical antibiotic prophylaxis guidelines and to evaluate the adherence of physicians to the guidelines.

## Methods

### Study design, setting and population

The study was conducted at the in-patient wards of a secondary care government hospital in Ras Al Khaimah, UAE from October 2017 to June 2018, after the approval of the Research and Ethics Committee of Ras Al Khaimah Medical and Health Sciences University (RAKMHSU REC) and Ras Al Khaimah Research and Ethics Committee (RAKREC) (Study No. MOHP/RAK/SUBC/NO: 42-2018-PG-P). Individual informed consent was waived for the conduct of this study by RAKMHSU REC and RAKREC as it was deemed a surveillance activity. It was a retrospective and prospective patient record review study using data collection forms for documenting data of patients who underwent surgical procedures during prior and post implementation of local hospital guidelines’ periods. During the study period, the hospital had a capacity of around 225 beds and it provided medical care in specialties of surgery, pediatric, urology, obstetrics, gynecology, dental, ear-nose-throat, ophthalmic and critical care including intensive care unit (ICU), neonatal intensive care unit (NICU) and special care baby unit (SCBU). ICU contains 8 beds and provides critical care services for adult patients, while NICU has 8 beds and provides critical care services for neonates. SCBU consists of 10 beds and provides medical care for neonates who are off ventilator. The hospital is used as one of the teaching centers for the medical, pharmacy and nursing undergraduate students of Ras Al Khaimah and other Emirates of UAE.

In ours study, the surgical wounds were categorized as class I (clean wounds) and class II (clean- contaminated wounds). All patients of either gender and of all ages who were admitted to the in-patient wards and underwent clean or clean-contaminated major surgical procedures in operating theatre were included in the study^14^. We excluded the patients who attended the outpatient department, patients who had a documented infection at the time of surgery as evaluated by onsite physician, patients with contaminated and dirty wounds and who underwent minor procedures in day care unit and not the major surgical procedures in main operating theater. During the period of data collection, 509 patients underwent surgery, out of which 16 patients were excluded from analysis based on exclusion criteria and 493 patients were enrolled in the present study. A unique study- specific number was assigned to each patient and any data leading to the personal identification of the patient were not collected. All methods were performed in accordance with the relevant guidelines and regulations.

### Implementation of antibiotic stewardship programme

In February 2018, a nominated hospital Antibiotic Stewardship Committee (ASPC) comprised of a multidisciplinary team including surgeon, anesthetist, pediatrician, clinical pharmacist, clinical microbiologist, infection control practitioner and nurses was created. ASPC reviewed international guidelines and adapted content from the Infectious Diseases Society of America (IDSA), Society for Healthcare Epidemiology of America (SHEA), American Society of Health System Pharmacists (ASHP) and the Centers for Disease Control and Prevention (CDC)^15–18^. Accordingly, the team designed the local hospital guidelines for surgical antibiotic prophylaxis of clean and clean- contaminated surgical procedures considering local microbiology data and released its first edition on April, 2018 (Tables 1, 2). The guidelines were made accessible on the hospital intranet. The clinical pharmacist played an important role in designing and implementing the hospital’s guidelines. The designing phase of the clinical guideline, was done by extensive reviewing of the scientific literature, documenting the current clinical and pharmaceutical processes in the hospital and by conducting several meetings with other members of ASPC. Additionally, the clinical pharmacist shared the evidence based clinical practices with each surgical team and collaborated with them to develope hospital antibiotic surgical prophylaxis guidelines with other ASPC members. Moreover, the clinical pharmacist played an essential role in the guidelines implementation phase by following the guideline’s approval process with the quality department and by composing educational materials and conducting educational lectures during the study period. The materials were also communicated by the clinical pharmacist to all the medical staff including head of departments and physicians and the guidelines were made accessible on the hospital intranet. In addition, official reminder emails were sent by the clinical pharmacist to physicians in order to encourage adherence to guideline.

**Table 1:** Preoperative antibiotics prophylaxis for the most commonly performed surgical procedures.

Surgical Procedure	Antibiotic		Alternative for Penicillin and / or Cephalosporin Allergy
	First Line	Second Line	
**Gastrointestinal and Abdomen**			
Bariatric involving gastroduodenal	Cefuroxime IV^$^ 1.5 g	Co-amoxiclav IV 1.2 g	♦Clindamycin IV 600 mg + Gentamycin IV 2 mg/Kg (Maximum: 320 mg) ♦Clindamycin IV 600 mg + Ciprofloxacin IV 400 mg
Biliary tract Open procedure			
Laparoscopic procedure (High-risk)*			
Bariatric involving Ilea	Cefuroxime IV 1.5 g + Metronidazole IV 500 mg	Co-amoxiclav IV 1.2 g + Metronidazole IV 500 mg	♦Metronidazole 500 mg + Gentamycin IV 2 mg/Kg (Maximum: 320 mg) ♦Metronidazole IV 500 mg + Ciprofloxacin IV 400 mg
Appendectomy for uncomplicated appendicitis			
Small intestine Obstructed			
**Gynecological**			
Cesarean delivery	Cefuroxime IV 1.5 g	♦Co-amoxiclav IV 1.2 g	Clindamycin IV 600 mg + Metronidazole 500 mg + Gentamycin 2 mg/Kg (Maximum: 320 mg)
Hysterectomy (vaginal or abdominal)	Cefuroxime IV 1.5 g	Co-amoxiclav IV 1.2 g	
Laparascopic abdominal vaginal hysterectomy			
Assistant delivery if GBS positive	Penicillin G (Benzylpenicillin) IV 3 g initially then 1.2 g IV every four hourly until delivery	Ampicillin, 2 g IV initial dose, then 1 g IV every 4 h until delivery	♦Clindamycin IV 900 mg, 8 hourly until delivery ♦Vancomycin IV 1 g, every 12 h until delivery ♦Clindamycin IV 600 mg + metronidazole IV 500 mg ♦Clarithromycin IV 500 mg + Metronidazole IV 500 mg
Perineal tear third/fourth degree	Cefuroxime IV 1.5 g + Metronidazole IV 500 mg	Co-amoxiclav IV 1.2 g + Metronidazole IV 500 mg	
Manual removal of the placenta			
**Urogenital**			
Lower tract instrumentation with risk factors for infection (includes transrectal prostate biopsy) TURP , TURBT	Ciprofloxacin IV 400 mg + Clindamycin IV 600 mg	Levofloxacin IV 500 mg + Clindamycin IV 600 mg	
Urethrotomy (Low risk)	Cefuroxime IV 1.5 g	Co-amoxiclav IV 1.2 g	Gentamycin IV 2 mg/kg (Maximum: 320 mg)
Urethrotomy (high risk)	Gentamicin IV 2 mg/kg + Teicoplanin IV 600 mg	♦Gentamycin IV 2 mg/kg (Maximum: 320 mg) + Clindamycin I.V 600 mg ♦Ciprofloxacin IV 400 mg + Clindamycin IV 600 mg ♦Levofloxacin IV 500 mg + Clindamycin IV 600 mg	
Clean with entry into urinary tract	Cefuroxime IV 1.5 g + Gentamycin IV 2 mg/Kg (Maximum: 320 mg)	Cefuroxime IV 1.5 g + Ciprofloxacin IV 400 mg	Gentamycin IV 2 mg/Kg (Maximum: 320 mg) + Clindamycin IV 600 mg
Clean without entry into urinary tract (High risk)			
**Ophthalmic**			
Cataract	Tobramycin 0.3% eye drop	♦Moxifloxacin 0.5% eye drop ♦Ciprofloxacin 0.3% eye drop ♦Ofloxacin 0.3% eye drop	
Glaucoma			
Orthopedic			
Clean operations involving hand, knee, or foot and not involving implantation of foreign materials	Co-amoxiclav IV 1.2 g	Cefuroxime IV 1.5 g	Clindamycin IV 600 mg + Gentamycin IV 2 mg/kg (Maximum: 320 mg)
Implantation of internal fixation devices (e.g., nails, screws, plates, wires)			
Total joint replacement			
**Thoracic**			
Non cardiac procedures, including lobectomy, pneumonectomy, lung resection, and thoracotomy	Cefuroxime IV 1.5 g	Co-amoxiclav IV 1.2 g + Gentamycin IV 2 mg/Kg (Maximum: 320 mg)	Clindamycin IV 600 mg Vancomycin IV 15 mg/kg (Maximum: 2 g)
Video-assisted thoracoscopic surgery	None	None	None
Oesophagoscopy, bronchoscopy ± intervention	None	None	None

^$^Intravenous route of administration. *Emergency procedures, diabetes, long procedure duration, intraoperative gallbladder rupture, age of > 70 years, conversion from laparoscopic to open cholecystectomy, American Society of Anesthesiologists classification of 3 or greater, episode of colic within 30 days before the procedure, intervention in less than one month for noninfectious complication, acute cholecystitis, bile spillage, jaundice, pregnancy, nonfunctioning gallbladder, immunosuppression, and insertion of prosthetic device.

**Table 2:** Recommended dose, administration and re-dosing interval of intravenous surgical prophylaxis antibiotics.

Antibiotic (Injection)	Adult Dose	Paediatric Dose*	Administration	Recommended Re-dosing Interval (from Initiation of Pre-operative Dose)
Ampicillin	2 g	50 mg/kg	Bolus over 3 to 5 min	2 h
Cefuroxime	1.5 g	50 mg/kg	Bolus over 3 to 5 min	4 h
Cefotaxime	1 g	50 mg/kg	Bolus over 3 to 5 min	3 h
Ceftriaxone	2 g	50–75 mg/kg	Infuse over 30 min	–
Ceftazidime	50 mg/kg	30—50 mg/kg (Maximum 6 g/day)	Bolus over 3 to 5 min	5 h
Ciprofloxacin	400 mg	10 mg/kg	Infused slowly into a large vein over 60 min	–
Clarithromycin	500 mg	7.5 mg/kg ( Maximum 500 mg)	Infuse over 60 min into large proximal vein	4 h (250 mg)
Clindamycin	600 mg	10 mg/kg	Infuse over at least 30 min	6 h
Co- amoxiclav	1.2 g	30 mg/kg (Maximum 1.2 g)	Bolus over 3 to 4 min	4 h
Gentamicin^§^	2–5 mg/kg based on dosing weight (single dose)	2.5 mg/kg based on dosing weight (1 month to 10yrs: Maximum 320 mg) (> 10 years: Maximum 560 mg)	Infuse over 30 min	–
Levofloxacin	500 mg	10 mg/kg	If volume 100 ml infuse over 60 min	–
Metronidazole	500 mg	15 mg/kg (Neonates weighing < 1200 g should receive a single 7.5-mg/kg/dose) ( Maximum: 500 mg)	Infuse over 20 min	8 h
Vancomycin^¥^	15 mg/kg	15 mg/kg ( Maximum: 1.5 g)	Infuse over 60—120 min (Administer ≤ 1 g over at least 60 min, > 1 g-1.5 g over at least 90 min, and > 1.5 g over 120 min)	–
Teicoplanin	600 – 800 mg	10 mg/kg ( Maximum: 400 mg)	Bolus over 3 to 5 min	–

*The Maximum pediatric dose should not exceed the usual adult dose.

^§^Dosing is based on the patient’s actual body weight. If the patient’s actual weight is more than 20% above ideal body weight (IBW), the dosing weight (DW) can be determined as follows: DW = IBW + 0.4 (actual weight − IBW). Ideal Body Weight (IBW) = 45.4 + [0.89 x (height {cm} − 152.4)] (+ 4.5 if male).

^¥^Vancomycin dose should be based on total body weight, rounded to the nearest 250 mg up to a maximum 2 g/dose.

The released guidelines described hospital antibiotic skin test policy, which emphasized on conducting intradermal skin test for β-lactam antibiotics such as pencillins, cephalosporins and carbapenems. The skin test was not recommended if a previous administration of that specific β-lactam antibiotic was uneventful. Testing was performed in the inner volar aspect of the forearm, with injection of 0.2 ml of diluted antibiotic solution as per manufacturer’s recommendation. The occurrence of any reaction was observed for 15–20 min and rated as positive or negative. A positive reaction is considered if 2 out of 3 of the following are present: a wheal of a diameter measuring at least ≥ 3 mm more than the negative control, itching sensation and a flare. The guidelines also indicated that the first dose of all intravenous medications which the patient will be receiving for the first time should be administered by provocative test administration under close observation and monitoring. The guidelines also focused on selection and administration of the appropriate antibiotic for surgical prophylaxis depending on the type of surgical procedure, site and risk for developing surgical site infections. In addition, the individual risk factors for every patient such as presence of prostheses, urinary catheters or stents, allergies, malignancy, diabetes, etc. were considered and the need for prophylaxis, the choice or dose of antibiotic was evaluated prior to administration. The guidelines instructed the physicians to avoid the use of broad spectrum antibiotics such as pipercillin/tazobactam, meropenem, etc. and restrain the use of third generation cephalosporin for surgical prophylaxis as they are associated with increased emerge of extended-spectrum β-lactamases and *Clostridioides difficile* strains. The guidelines also recommend the use of gentamicin rather than amikacin for preoperative prophylaxis in order to maintain the very low resistance pattern to amikacin in the hospital. This recommendation is based on the local hospital antibiogram at the time of study, which demonstrated higher sensitivity results of amikacin toward Gram negative bacteria in comparison to gentamicin. For example, the sensitivity toward *Escherichia coli* which was the predominant organism in the hospital with number of isolates exceeding 250, were 98.9% for amikacin versus 85% for gentamicin. First and second generation cephalosporins and amoxicillin/clavulanic acid were the first drugs of choice for majority of the surgical procedures. As per the guidelines, all prophylactic antibiotics should be intended for administration within 60 min before skin incision, with the exception of vancomycin and ciprofloxacin that should be given within 60- 120 min of skin incision. The use of antibiotic prophylaxis for more than 24 h is not recommended for most procedures, except in special circumstances such as bowel perforation or active infection discovered during the procedure. Re-dosing of antibiotic for patients who experience major blood loss (greater than 1500 ml) or for operations lasting > 4 h should be reconsidered and re-dosing interval/ dose for adults and pediatrics should be adjusted accordingly.

The dosages of surgical prophylaxis were included in the guidelines. All antibiotics which are mentioned in local hospital guidelines were included in the hospital Drug Formulary as part of Ministry of Health and Prevention Drug Formulary Management System^19^.

### Data collection and outcome measures

The two months’ data, from October to November 2017 was collected retrospectively for patients who underwent surgical procedures prior to implementation of local hospital guidelines, while post-implementation data was collected prospectively from April to May 2018. The patient details were collected from the electronic medical records. All the relevant details pertaining to patient’s demographics, medical and medication history, surgery type and date, length of hospital stay, antibiotics skin sensitivity test, prescription characteristics such as the generic and proprietary name of the drug, its type (generation), indication for antibiotic use, route, dose and duration of antibiotic therapy, common adverse drug reactions encountered and other details were obtained through the electronic medical records system and documented into approved and authorized data collection forms. The collected data were entered in Microsoft Excel 2013 sheets for primary data segregation and analyzed for average, standard deviation and percentages.

The outcome measures were the change in prescribing practice of surgical antibiotic prophylaxis before and after the launch of local surgical prophylaxis guidelines for the following parameters: selection of antibiotics for surgical prophylaxis, administration of antibiotic within 60 min before incision (60–120 min for vancomycin & ciprofloxacin) and discontinuation of antibiotic prophylaxis within 24 h after completion of surgery.

### Data analysis

Descriptive statistical analysis for continuous variables was done with Statistical Package for the Social Sciences software, version 20.0 (IBM Corp. Released 2011, IBM SPSS Statistics for windows version 20.0, Armonk, NY: USA) and Graph Pad Software version prism 7 (Graph Pad Software Inc., 2017). Results were presented in the form of text and tables. Data was summarized by Mean ± SD for continuous variables and percentage (%) for categorical variables. The comparison between prior and post local hospital guidelines implementation was done by unpaired t-test/Mann Whitney U test for continuous variables and Chi-Square test/Fisher’s exact test for categorical variables. All p-values less than 0.05 were deemed as statistically significant.

### Ethical approval and consent to participate

The study was approved by the Research and Ethics Committees of RAK Medical and Health Sciences University and the Emirate of Ras Al Khaimah (Study No. MOHP/RAK/SUBC/NO: 42-2018-PG-P). Individual informed consent was waived for the conduct of this study by these committees as it was deemed a surveillance activity.

## Results

Surgical prophylactic antibiotics were administered to 493 patients, out of which 347 patients’ data were included in pre-implementation period and post-implementation period included 146 patients. Both periods showed similar results in terms of sex, age, nationality and body weight distribution, with an overall female predominance, major age group of 31–40 years, UAE nationality and body weight group of 61–80 kg predominance.

Among the 493 patients, 28.8% and 10.9% patients enrolled in pre-implementation period and post-implementation period respectively, had one or two co-morbid conditions such as diabetes mellitus, hypertension, hypothyroidism, ischemic heart diseases, chronic obstructive pulmonary disease, asthma and other medical conditions than the above mentioned ones. Antibiotics skin sensitivity test revealed no significant difference between prior and post local hospital guidelines implementation periods (Fisher’s exact test; *P* > 0.05). No allergies were documented for food or antibiotics.

A significant difference between the wound classes was observed in the pre-implementation and post-implementation periods: clean wounds were 255 (point estimate 95% CI 0.7349) in pre-implementation period and 81 (point estimate 95% CI 0.5548) in post-implementation period. Clean-contaminated wounds were 92 (point estimate 95% CI 0.2677) in pre-implementation period and 65 (point estimate 95% CI 0.4455) in post-implementation period (Fisher’s exact test; *P* = 0.0001).

The study included 14 defined categories of surgical procedures and one non-defined (other) category. Patients’ and procedures’ characteristics in pre and post-implementation periods are displayed in Table 3.

**Table 3:** Patient and procedure characteristics in pre and post-implementation periods.

Patient and Procedure Characteristics	Pre-implementation Period (n = 347) *N* (%)	Point Estimate 95% CI	Post-implementation Period (n = 146) *N* (%)	Point Estimate 95% CI
Gender (Female)^¥^	216 (62.2)	0.6225	99 (67.8)	0.6781
Age in years (± SD)	34.7** ± **19.2	32.68, 36.72^a^	27.5** ± **14.5	25.148, 29.852^a^
Nationality (UAE Nationals) ^¥^	193 (55.6)	0.5562	93 (63.7)	0.6370
Body weight (61–80 kg) ^¥^	144 (41.5)	0.4159	57 (39.0)	0.3932
Co-morbidities (Diabetes mellitus) ^¥^	61 (17.6)	0.1793	9 (6.1)	0.0729
Antibiotic skin sensitivity test	244 (70.3)	0.7032	110 (75.3)	0.7534
Wound type Clean	255 (73.5)	0.7349	81 (55.5)	0.5548
Clean-contaminated	92 (26.5)	0.2677	65 (44.5)	0.4455
Surgical procedures Appendectomy	13 (3.8)	0.0425	20 (13.7)	0.1463
Bariatric	–	–	6 (4.1)	0.0529
Cesarean delivery	106 (30.5)	0.3076	67 (45.9)	0.4600
Gastrointestinal	3 (0.8)	0.0140	2 (1.3)	0.0262
Gynecologic	33 (9.5)	0.0995	9 (6.1)	0.0729
Head and neck	1 (0.3)	0.0083	–	–
Hernia	6 (1.7)	0.0226	2 (1.3)	0.0262
Neurosurgery	1 (0.3)	0.0083	1 (0.6)	0.0195
Ophthalmic	31 (8.9)	0.0938	4 (2.7)	0.0395
Orthopedic	26 (7.5)	0.0796	8 (5.4)	0.0662
Plastic surgery	12 (3.4)	0.0397	3 (2.1)	0.0328
Thoracic	17 (4.9)	0.0539	2 (1.3)	0.0262
Urologic	21 (6.0)	0.0653	15 (10.3)	0.1129
Vascular	6 (1.7)	0.226	–	–
Other	71 (20.4)	0.2078	7 (4.8)	0.0595

^¥^Major characteristic.

^a^95% confidence interval (CI) calculated using Z statistics.

The antibiotic stewardship committee in general and the clinical pharmacist in particular carried out the analysis of prescriptions adequacy in accordance with the international and local hospital guidelines. The results showed that all the surgical prophylactic medications were prescribed by their generic names. The most frequently used antibiotic in pre-implementation period was cefuroxime (110; 34%), followed by amoxicillin- clavulanic acid (84; 20.8%). Similarly, cefuroxime (68; 86.2%) was frequently administered in post-implementation period. This was followed by ceftriaxone (8; 10.1%). The second and third generation cephalosporins were the most widely administered antibiotics accounting for 250 (62.1%) of surgical prophylactic medications, out of which 174 (69.6%) was administered in in pre-implementation period. This was decreased to 76 (30. 4%) in post- implementation period where the third generation ceftriaxone was less administered and the second generation cefprozil was not administered (Table 4). Majority (353; 87.6%) of antibiotics were administered via intravenous (IV) route of administration as per the guidelines. The dose was appropriate for all the surgical procedures and ranged from 50 mg to 4.5 gm.

**Table 4:** Prophylactic antibiotics in surgical procedures.

Antibiotics	Pre-implementation Period		Post- implementation Period		Total	
	*N*	%	*N*	%	*N*	%
Cefuroxime	110	34	68	86.2	178	44.3
Amoxicillin-clavulanic acid	84	26	–	–	84	20.8
Ceftriaxone	53	16.4	8	10.1	61	15.1
Ciprofloxacin	22	6.8	–	–	22	5. 5
Cefprozil	11	3.4	–	–	11	2.7
Piperacillin/tazobactam	8	2.4	–	–	8	2
Nitrofurantoin	6	2	1	1.3	7	1.7
Clarithromycin	6	2	–	–	6	1.5
Levofloxacin	4	1.2	–	–	4	1
Vancomycin	3	0.9	–	–	3	0.7
Clindamycin	2	0.6	1	1.3	3	0.7
Amikacin	2	0.6	1	1.3	3	0.7
Linezolid	2	0.6	–	–	2	0.5
Metronidazole	2	0.6	–	–	2	0.5
Other*	9	2.5	–	–	9	2.3
Total	324	100	79	100	403	100

*Tigecycline, gentamicin, oloxacin, ceftazidime, moxifloxacin, azithromycin, cefixime and norfloxacin.

Antibiotics in pre-implementation period were mainly administered for 3 days (114; 32.9%). The rest were administered for 5 days (89; 25.7%) and 7 days (41; 11.8%). Similarly, majority of surgical prophylactic antibiotics in post-implementation period were administered for 3 days (118; 80.8%). This was followed by administration for 5 days (10; 6.8%) and 7 days (6; 4.1%) respectively. The average duration of therapy for cefuroxime was 3.8 ± 1.2 days in pre-implementation period which was reduced to 2.6 ± 0.6 in post-implementation period. The duration of hospitalization in pre implementation period ranged from 1 to 59 days and it was slightly higher (*p* > 0.05) than post implementation period which ranged from 1 to 27 days. The mean length of hospital stay was 5.311 ± 6.021 days (95% CI 4.67749, 5.94451) in pre- implementation period as compared to 4.904 ± 3.716 days (95% CI 4.30124, 5.50676) in post- implementation period. No adverse drug reaction was observed during the study period.

Table 5 shows the compliance of physicians to the local hospital guidelines. The comparison was considered for the antibiotic choice, timing and discontinuation of prophylaxis. The results revealed the non-significant difference (*p* = 0.552) in the selection of surgical prophylactic antibiotics by physicians between pre-implementation and post-implementation periods. Similarly, the rate of compliance to the timing of administration of the prophylactic antibiotic one hour before incision between the two periods was non-significant (*P* = 0.061). Whilst data for the adherence of physicians to the optimal post-operative duration of treatment revealed a significant difference (*P* < 0.00001) between the pre-implementation and post-implementation periods. The noncompliance with the discontinuation of antibiotics was observed in 244 (70. 3%; point estimate 95% CI 0.7032) cases in pre-implementation period where antibiotics were discontinued after 3, 5 and 7 days. The noncompliance was further observed in 134 (91.7%; point estimate 95% CI 0.9122) cases in post-implementation period.

**Table 5:** Comparison of physicians’ compliance to antimicrobial prophylaxis guidelines pre and post the implementation.

Parameters	Pre-implementation Period (n = 347)		Point Estimate 95% CI	Post-implementation Period (n = 146)		Point Estimate 95% CI	*P* Value
	N	%		N	%		
**Selection of antibiotic**							
1.1 Appropriate selection	192	55.3	0.5533	76	52.1	0.5205	0.552
1.2 Inappropriate selection	155	44.7	0.4473	70	47.9	0.4800	
**Optimal timing of the first dose**^**§**^							
2.1 Compliance to timing of administration	177	51	0.5101	88	60.3	0.6027	0.061
2.2 Non- compliance to timing of administration	170	49	0.4900	58	39.7	0.3999	
**Optimal postoperative duration**^**§§**^							
3.1 Compliance to discontinuation timing	103	29.7	0.2991	12	8.3	0.0929	< 0.00001
3.2 Non-compliance to discontinuation timing	244	70.3	0.7032	134	91.7	0.9122	
Total compliance	472	45.3^¥^		176	40.2		0.08

^§^Administration of surgical prophylaxis medications within 1 h before incision or within 2 h for ciprofloxacin & vancomycin.

^§§^Discontinuation of surgical prophylaxis within 24 h after surgery.

^¥^Fisher’s exact test was used for comparing of data before and after the implementation of guidelines. *p* < 0.05 was considered statistically significant.

Total compliance was calculated as the percentage of compliance in selection, administration and discontinuation of surgical prophylactic antibiotics. It decreased from 45.3% pre-implementation value to 40.2% post- implementation value (*P* = 0.08), mainly due to non-compliance to discontinuation timing.

## Discussion

The appropriate implementation of an antibiotics stewardship program in a secondary care hospital is often challenging, given the involvement of various medical specialties with different department structures. The present study is one of the few studies that reports the implementation of antibiotic stewardship program in UAE and the Gulf region. It provides information of the current prescribing practice of antibiotic surgical prophylaxis in Ras Al Khaimah and the results of this study can serve as a baseline for future comparison.

Antibiotic selection is influenced by the type of organism causing wound infection in the specific surgical procedure and by the relative costs of available medications. Majority of the wounds in our study were clean and we observed that β-lactam class of antibiotics were the most commonly administered drugs in the hospital. The current study site is a government hospital and all Emirati nationals have access to free medical treatment^20^. In addition, Ras Al-Khaimah differs from the other emirates in that UAE nationals have maintained majority of the population of the country making up to 50 percent of the population as compared to other emirates of UAE where nationals make 20 percent of the total population^21^. Similarly, majority of the data collected in this study shows the predominance of UAE nationality in Ras Al-Khaimah.

In accordance with the international and local hospital guidelines, cephalosporins/penicillins were appropriately selected and administered than other classes of antibiotics. However, the first generation injectable cephalosporins such as cefazolin were not administered frequently during the study period. Cefazolin was a non-formulary medication which was not listed in hospital formulary list during the study period, therefore, the second generation cefuroxime was the most commonly prescribed antibiotic in both the periods, and has definitely became the first choice in post implementation period. Similar results were also reported by Garcell et al*.* in which cefuroxime was the most frequently used drug in a hospital in Qatar^22^. The broad activity of cefuroxime against the susceptible microorganisms in the region and the surrounding countries, in addition to its proven clinical effectiveness and tolerability could have attributed to its preference as one of the most commonly used surgical prophylactic antibiotic. The appropriate selection of surgical prophylactic antibiotics in the present study (55.3%; point estimate 95% CI 0.5533) pre and 52.1% ; point estimate 95% CI 0.5205 post implementation) was almost comparable to that of an Australian study (53.3%) conducted by Bull et al.^23^ but lesser to that reported by Pittalis et al. (84.5%)^24^.

Adequate antibiotic administration timing is critical to the effectiveness of surgical prophylaxis and the optimal timing of the first dose in particular, plays an important role in maintaining adequate tissue concentrations of the administered antibiotic at the time of the incision and throughout the surgical procedure^25–27^. Based on the various reports, the optimal timing varied from 30 to 120 min depending on the half-life and protein binding of the administered antibiotics and the underlying condition of the individual patient^26,28–30^. In addition, non-adherence to recommended timing intervals is reported in various studies^24,31,32^. In our study, we observed an improvement in compliance of physicians to the administration of antibiotics from 51%; point estimate 95% CI 0.5101 in pre- implementation period to 60.3%; point estimate 95% CI 0.6027 in post- implementation period. Similarly, other studies reported an increasing in compliance rate to surgical antibiotic prophylaxis as a result of guidelines implementation^33–35^.

Prolonged administration of antibiotics for surgical procedures beyond the 24 h is frequently practiced by physicians and many studies reported the same^36–39^. Although it was found that the risk of surgical site infections does not decrease, but instead, the prolonged administration promoted antimicrobial resistance and increased the incidence of antibiotic-associated complications^40,41^. Our study too showed that physicians did not discontinue the administration of antibiotic prophylaxis regimen within 24 h in many surgical procedures and instead, they administered the antibiotics for 3, 5 and 7 days before and after implementation of the guidelines. Their inclination towards continuing administration of antibiotics for several days after surgery was influenced by their own judgment for taking precaution against infection and by having mixed opinion regarding the appropriate duration of antibiotics administration based on earlier reported studies^42–45^. Thus, they were hesitant to administer short courses of antibiotics and preferred the longer ones. Consistent with the current study findings, Putnam et al*.* reported the inability in achieving adherence to guidelines after multiple interventions^46^. The same non-adherence was also observed in many earlier studies^10,11,32,47^.

The overall compliance rate to the guidelines was reported to be affected by many factors^48^, out of which the prolonged antibiotic administration contributed significantly to the noncompliance to the SAP guidelines^49–53^. We obtained comparable results, where the overall compliance rate decreased in the post implementation period from 45.3 to 40.2%, mainly due to non-adherence to surgical prophylaxis discontinuation timing. However, there is a potential for an improvement in the post-operative discontinuation timing of the antibiotic to < 24 h where applicable. The initiation of educational intervention via cyclic auditing strategy might play an important role in changing the preference of physicians towards the use of short courses of antibiotics in the hospital.

Many research studies reported the importance of the clinical pharmacist in improving the compliance rate of physicians to the guidelines^11,24,49,54^. Our experience during stewardship program implementation also confirmed the importance of involving a clinical pharmacist as part of the multidisciplinary team. The education of the hospital staff about the antimicrobial prophylaxis in surgery and the accurate implementation of the program was carried out mainly by the clinical pharmacist who played a major role in optimizing antimicrobial treatment and in promoting rational utilization of surgical prophylactic antibiotics.

This study provides valuable information regarding the antibiotic surgical prophylaxis practice in UAE, in particular in the emirate of Ras Al Khaimah, since this is the first study to evaluate the guidelines implementation and adherence of physicians to these guidelines. However, a number of limitations should be noted. The results of this study cannot be generalized since this was a single center study and reveals the practice at a single site. Furthermore, the study conducted using a small sample size of patients as the data collection period was only for a short duration (2 months prior and 2 months post guidelines implementation). No control group was included in the study, therefore, there is a possibility of confounding considering the likelihood for seasonal trends in antibiotic use.

## Conclusion

Our study highlighted the multidisciplinary approach followed in Ras Al Khaimah government hospital to design and implement the surgical antimicrobial prophylaxis guidelines. Although in its budding stage, it establishes the first step in overcoming the antibiotic resistance in the emirate. The decreased overall compliance rate in the post implementation period was most commonly due to the use of antimicrobial prophylaxis for longer duration than stated in the guidelines. Therefore, we recommend the continuous medical education and cyclic auditing for improving the adherence.

## Data Availability

The data analyzed during this study are included in this published article. The basic datasets generated during the current study are available upon request to the corresponding author.

## References

[1] WHO AMR Report, 2014–World Health Organization. *Antimicrobial Resistance: Global Report on Surveillance*. http://www.who.int/drugresistance/documents/surveillancereport/en/. (2014) Accessed 2 December 2019.

[2] Centers for Disease Control and Prevention. *Antibiotic/Antimicrobial Resistance (AR/ MR)*. https://www.cdc.gov/drugresistance/about.html. (2018) Accessed 15 April 2020.

[3] Harbarth S (2015). Antimicrobial resistance: one world, one fight. Antimicrob. Resist Infect. Control.

[4] Balkhy H (2016). The strategic plan for combating antimicrobial resistance in Gulf Cooperation Council States. J. Infect. Public Health.

[5] Al-Dhaheri AS, Al-Niyadi MS, Al-Dhaheri AD, Bastaki SM (2009). Resistance patterns of bacterial isolates to antimicrobials from 3 hospitals in the United Arab Emirates. Saudi Med. J..

[6] Al-Zarouni M, Senok A, Rashid F, Al-Jesmi SM, Panigrahi D (2008). Prevalence and antimicrobial susceptibility pattern of extended-spectrum beta-lactamase-producing Enterobacteriaceae in the United Arab Emirates. Med. Princ. Pract..

[7] Rotimi VO, Jamal W, Pal T, Sonnevend A, Dimitrov TS, Albert MJ (2008). Emergence of multidrug-resistant Salmonella spp and isolates with reduced susceptibility to ciprofloxacin in Kuwait and the United Arab Emirates. Diagn. Microbiol. Infect. Dis..

[8] Najiba, M. A. United Arab Emirates Ministry of Health and Prevention. UAE Higher Committee on AMR. UAE National Action Plan for AMR. http://www.icamr-uae.com/presentations/national-amr-plan-wide.pdf. Accessed on 17 May 2020.

[9] Khanem JA, Khair H, Benson R (2012). Antibiotic prophylaxis for caesarean section at Tawam Hospital, UAE. Gulf Med. J..

[10] Abu-Gharbieh E, Fahmy S (2012). Adherence to surgical site infection guidelines in cardiac surgery in a tertiary hospital in Dubai, United Arab Emirates. Trop J. Pharm. Res..

[11] El-Hassan M, Elnour AA, Farah HH, Shehab A, Al Kalbani NM, Asim S (2015). Clinical pharmacists’ review of surgical antimicrobial prophylaxis in a tertiary hospital in Abu Dhabi. Int. J. Clin. Pharm..

[12] Nasr Z, Paravattil B, Wilby KY (2017). The impact of antimicrobial stewardship strategies on antibiotic appropriateness and prescribing behaviours in selected countries in the Middle East: a systematic review. Eastern Medit. Health J..

[13] Alghamdi S, Shebl NA, Aslanpour Z, Shibl A, Berrou I (2018). Hospital adoption of antimicrobial stewardship programmes in Gulf Cooperation Council countries: A review of existing evidence. J. Global Antimicrob. Res..

[14] Raosoft® software Sample size calculator http://www.raosoft.com/samplesize.html. Accessed 29 Aug 2017.

[15] Dellit TH, Owens RC, McGowan JE (2007). Infectious Diseases Society of America and the Society for Healthcare Epidemiology of America guidelines for developing an institutional program to enhance antimicrobial stewardship. Clin. Infect. Dis..

[16] Pope SD, Dellit TH, Owens RC, Hooton TM (2009). Results of survey on implementation of Infectious Diseases Society of America and Society for Healthcare Epidemiology of America guidelines for developing an institutional program to enhance antimicrobial stewardship. Infect. Control Hosp. Epidemiol..

[17] Barlam TF, Cosgrove SE, Abbo LM (2016). Executive summary: implementing an antibiotic stewardship program: Guidelines by the Infectious Diseases Society of America and the Society for Healthcare Epidemiology of America. Clin. Infect. Dis..

[18] Pollack LA, Srinivasan A (2014). Core elements of hospital antibiotic stewardship programs from the Centers for Disease Control and Prevention. Clin. Infect. Dis..

[19] United Arab Emirates Ministry of Health and Prevention. *Formulary Management System*. https://www.mohap.gov.ae/en/services/Pages/201.aspx. Accessed 21 March 2020.

[20] Good health and well-being - The official portal of the UAE Government. *Goverment support of health sector*. https://u.ae/en/about-the-uae/leaving-no-one-behind/3goodhealthandwellbeing (2020). Accessed 23 Dec 2020.

[21] Ras al-Khaimah Information. *UAE medical insurance*. https://www.uae-medical-insurance.com/resources/uae-topics/ras-al-khaimah/ (2018). Accessed 8 Dec 2019.

[22] Humberto G (2017). Impact of a focused antimicrobial stewardship program in adherence to antibiotic prophylaxis and antimicrobial consumption in appendectomies. J. Inf. Public Health.

[23] Bull, A. L. *et al*. Compliance with surgical antibiotic prophylaxis: reporting from a statewide surveillance programme in Victoria, Australia. *J. Hosp. Infect*.** 63**, 140–147 (2006). 10.1016/j.jhin.2006.01.018 16621135

[24] Pittalis, S. *et al*. Appropriateness of surgical antimicrobial prophylaxis in the Latium region of Italy, 2008: a multicenter study. *Surg. Infect. (Larchmt)* **14**, 381–384 (2013). 10.1089/sur.2012.189 23848414

[25] Zelenitsky SA, Ariano RE, Harding GKM (2002). Antibiotic pharmacodynamics in surgical prophylaxis: An association between intraoperative antibiotic concentrations and efficacy. Antimicrob. Agents Chemother..

[26] de Jonge SW, Gans SL, Atema JJ, Solomkin JS, Dellinger PE, Boermeester MA (2017). Timing of preoperative antibiotic prophylaxis in 54 552 patients and the risk of surgical site infection: a systematic review and meta-analysis. Medicine (Baltimore).

[27] Classen DC, Evans RS, Pestotnik SL (1992). The timing of prophylactic administration of antibiotics and the risk of surgical-wound infection. N. Engl. J. Med..

[28] Steinberg JP, Braun BI, Hellinger WC (2009). Timing of antimicrobial prophylaxis and the risk of surgical site infections: results from the Trial to Reduce Antimicrobial Prophylaxis Errors. Ann. Surg..

[29] Ho VP, Barie PS, Stein SL (2011). Antibiotic regimen and the timing of prophylaxis are important for reducing surgical site infection after elective abdominal colorectal surgery. Surg. Infect. (Larchmt).

[30] van Kasteren ME, Mannien J, Ott A (2007). Antibiotic prophylaxis and the risk of surgical site infections following total hip arthroplasty: timely administration is the most important factor. Clin. Infect. Dis..

[31] Musmar SM, Baba H, Owais A (2014). Adherence to guidelines of antibiotic prophylactic use in surgery: A prospective cohort study in North West Bank, Palestione. BMC Surg.

[32] Bratzler DW, Houck PM, Richards C, Steele L, Dellinger EP, Fry DE, Wright C, Ma A, Carr K, Red L (2005). Use of antimicrobial prophylaxis for major surgery: baseline results from the National Surgical Infection Prevention Project. Arch. Surg..

[33] Mahmoudi L, Ghouchani M, Mahi-Birjand M, Bananzadeh A, Akbari A (2019). Optimizing compliance with surgical antimicrobial prophylaxis guidelines in patients undergoing gastrointestinal surgery at a referral teaching hospital in southern Iran: clinical and economic impact. Infect Drug Resist..

[34] Price J, Ekleberry A, Grover A (1999). Evaluation of clinical practice guidelines on outcome of infection in patients in the surgical intensive care unit. Crit. Care Med..

[35] So JP, Aleem IS, Tsang DS, Matlow AG, Wright JG, Surgical Site Infection Task Force (2015). Task Force Increasing compliance with an antibiotic prophylaxis guideline to prevent pediatric surgical site infection: Before and after study. Ann Surg..

[36] Baqain ZH, Hyde N, Patrikidou A, Harris M (2004). Antibiotic prophylaxis for orthognathic surgery: A prospective, randomised clinical trial. Br. J. Oral Maxillofac. Surg..

[37] Suzuki T, Sadahiro S, Maeda Y, Tanaka A, Okada K, Kamijo A (2011). Optimal duration of prophylactic antibiotic administration for elective colon cancer surgery: A randomized, clinical trial. Surgery..

[38] Regimbeau JM, Fuks D, Pautrat K, Mauvais F, Haccart V, Msika V (2014). Effect of postoperative antibiotic administration on postoperative infection following cholecystectomy for acute calculous cholecystitis: A randomized clinical trial. JAMA.

[39] Becker A, Koltun L, Sayfan J (2008). Impact of antimicrobial prophylaxis duration on wound infection in mesh repair of incisional hernia: Preliminary results of a prospective randomized trial. Eur. Surg..

[40] Harbarth S, Samore MH, Lichtenberg D (2000). Prolonged antibiotic prophylaxis after cardiovascular surgery and its effect on surgical site infections and antimicrobial resistance. Circulation.

[41] Suehiro T, Hirashita T, Araki S (2008). Prolonged antibiotic prophylaxis longer than 24 hours does not decrease surgical site infection after elective gastric and colorectal surgery. Hepatogastroenterology.

[42] Tamayo E, Gualis J, Florez S, Castrodeza J, Eiros Bouza JM, Alvarez FJ (2008). Comparative study of single-dose and 24-hour multiple-dose antibiotic prophylaxis for cardiac surgery. J. Thorac. Cardiovasc. Surg..

[43] Hall JC, Christiansen KJ, Goodman M (1998). Duration of antimicrobial prophylaxis in vascular surgery. Am. J. Surg..

[44] Fujita S, Saito N, Yamada T (2007). Randomized, multicenter trial of antibiotic prophylaxis in elective colorectal surgery: Single dose vs 3 doses of a second-generation cephalosporin without metronidazole and oral antibiotics. Arch. Surg..

[45] Bentley KC, Head TW, Aiello GA (1999). Antibiotic prophylaxis in orthognathic surgery: A 1-day versus 5-day regimen. J. Oral Maxillofac. Surg..

[46] Putnam LR, Chang CM, Rogers NB, Podolnick JM, Sakhuja S, Matusczcak M, Austin MT, Kao LS, Lally KP, Tsao K (2015). Adherence to surgical antibiotic prophylaxis remains a challenge despite multifaceted interventions. Surgery.

[47] Kobayashi M, Takesue Y, Kitagawa Y, Kusunoki M, Sumiyama Y (2011). Antimicrobial prophylaxis and colon preparation for colorectal surgery: Results of a questionnaire survey of 721 certified institutions in Japan. Surg. Today.

[48] Saied T, Hafez SF, Kandeel A, El-Kholy A, Ismail G, Aboushady M (2015). Antimicrobial stewardship to optimize the use of antimicrobials for surgical prophylaxis in Egypt: A multicenter pilot intervention study. Am. J. Infect. Control.

[49] Abdel-Aziz A, El-Menyar A, Al-Thani H, Zarour A, Parchani A, Asim M (2013). Adherence of surgeons to antimicrobial prophylaxis guidelines in a tertiary general hospital in a rapidly developing country. Adv. Pharmacol. Sci..

[50] Tourmousoglou CE, Yiannakopoulou EC, Kalapothaki V, Bramis J, Papadopoulos JS (2008). Adherence to guidelines for antibiotic prophylaxis in general surgery: a critical appraisal. J. Antimicrob. Chemother..

[51] Elbur AI, Yousif MA, Elsayed ASA, Abdel-Rahman ME (2013). An audit of prophylactic surgical antibiotic use in a Sudanese Teaching Hospital. Int. J. Clin. Pharm..

[52] Vessal G, Namazi S, Davarpanah MA, Foroughinia F (2011). Evaluation of prophylactic antibiotic administration at the surgical ward of a major referral hospital, Islamic Republic of Iran. East Mediterr. Health J..

[53] Alemkere G (2018). Antibiotic usage in surgical prophylaxis: a prospective observational study in the surgical ward of Nekemte Referral Hospital. PLoS ONE.

[54] Prado M, Lima M, Bergsten-Mendes G (2002). The implementation of a surgical antibiotic prophylaxis program: The pivotal contribution of the hospital pharmacy. Am. J. Infect. Control.

